# Supplementary material for: A Critical View of the Application of the APEX Software (Aqueous Photochemistry of Environmentally-Occurring Xenobiotics) to Predict Photoreaction Kinetics in Surface Freshwaters
Source: Molecules. 2019 Dec 18;25(1):9. doi: 10.3390/molecules25010009 (PMC7017383; doi:10.3390/molecules25010009)
Supplement: Supplementary file 1 [file molecules-25-00009-s001.zip › 3.2.4_gcc-4.4.0.zip!/3.2.4_gcc-4.4.0/3.2.4_gcc-4.4.0/doc/octave/html/interpreter/Calling-External-Code-from-Oct_002dFiles.html]

Calling External Code from Oct-Files - Untitled


Next: Allocating Local Memory in Oct-Files,
Previous: Calling Octave Functions from Oct-Files,
Up: Oct-Files


---

#### A.1.9 Calling External Code from Oct-Files

Linking external C code to Octave is relatively simple, as the C
functions can easily be called directly from C++. One possible issue is
the declarations of the external C functions might need to be explicitly
defined as C functions to the compiler. If the declarations of the
external C functions are in the header `foo.h`, then the manner in
which to ensure that the C++ compiler treats these declarations as C
code is

```
     #ifdef __cplusplus
     extern "C"
     {
     #endif
     #include "foo.h"
     #ifdef __cplusplus
     }  /* end extern "C" */
     #endif
```

Calling Fortran code however can pose some difficulties. This is due to
differences in the manner in compilers treat the linking of Fortran code
with C or C++ code. Octave supplies a number of macros that allow
consistent behavior across a number of compilers.

The underlying Fortran code should use the `XSTOPX` function to
replace the Fortran `STOP` function. `XSTOPX` uses the Octave
exception handler to treat failing cases in the fortran code
explicitly. Note that Octave supplies its own replacement blas
`XERBLA` function, which uses `XSTOPX`.

If the underlying code calls `XSTOPX`, then the `F77_XFCN`
macro should be used to call the underlying fortran function. The Fortran
exception state can then be checked with the global variable
`f77_exception_encountered`. If `XSTOPX` will not be called,
then the `F77_FCN` macro should be used instead to call the Fortran
code.

There is no harm in using `F77_XFCN` in all cases, except that for
Fortran code that is short running and executes a large number of times,
there is potentially an overhead in doing so. However, if `F77_FCN`
is used with code that calls `XSTOP`, Octave can generate a
segmentation fault.

An example of the inclusion of a Fortran function in an oct-file is
given in the following example, where the C++ wrapper is

```
```
     #include <octave/oct.h>
     #include <octave/f77-fcn.h>
     
     extern "C" 
     {
       F77_RET_T 
       F77_FUNC (fortsub, FORTSUB) 
             (const int&, double*, F77_CHAR_ARG_DECL  
              F77_CHAR_ARG_LEN_DECL);
     }
     
     DEFUN_DLD (fortdemo , args , , "Fortran Demo.")
     {
       octave_value_list retval;  
       int nargin = args.length();
       if (nargin != 1)
         print_usage ();
       else
         {
           NDArray a = args(0).array_value ();
           if (! error_state)
             {
               double *av = a.fortran_vec ();
               octave_idx_type na = a.nelem ();
               OCTAVE_LOCAL_BUFFER (char, ctmp, 128);
     
               F77_XFCN (fortsub, FORTSUB, (na, av, ctmp 
                         F77_CHAR_ARG_LEN (128)));
     
     	  retval(1) = std::string (ctmp);
     	  retval(0) = a;
             }
         }
       return retval;
     }
```
```

and the fortran function is

```
```
           subroutine fortsub (n, a, s)
           implicit none
           character*(*) s
           real*8 a(*)
           integer*4 i, n, ioerr
           do i = 1, n
             if (a(i) .eq. 0d0) then
               call xstopx ('fortsub: divide by zero')
             else
               a(i) = 1d0 / a(i)
             endif
           enddo
           write (unit = s, fmt = '(a,i3,a,a)', iostat = ioerr)
          $       'There are ', n,
          $       ' values in the input vector', char(0)
           if (ioerr .ne. 0) then
             call xstopx ('fortsub: error writing string')
           endif
           return
           end
```
```

This example demonstrates most of the features needed to link to an
external Fortran function, including passing arrays and strings, as well
as exception handling. An example of the behavior of this function is

```
     [b, s] = fortdemo (1:3)
     =>
       b = 1.00000   0.50000   0.33333
       s = There are   3 values in the input vector
     [b, s] = fortdemo(0:3)
     error: fortsub:divide by zero
     error: exception encountered in Fortran subroutine fortsub_
     error: fortdemo: error in fortran
```
